# Single-cell transcriptome atlas of peripheral immune features to Omicron breakthrough infection under booster vaccination strategies

**DOI:** 10.3389/fimmu.2024.1460442

**Published:** 2025-01-06

**Authors:** Yuwei Zhang, Shanshan Han, Qingshuai Sun, Tao Liu, Zixuan Wen, Mingxiao Yao, Shu Zhang, Qing Duan, Xiaomei Zhang, Bo Pang, Zengqiang Kou, Xiaolin Jiang

**Affiliations:** ^1^ Infectious Disease Prevention and Control Section, Shandong Center for Disease Control and Prevention, Jinan, Shandong, China; ^2^ School of Public Health and Health Management, Shandong First Medical University and Shandong Academy of Medical Sciences, Jinan, Shandong, China; ^3^ Department of Infectious Disease Control, Yantai Center for Disease Control and Prevention, Yantai, Shandong, China; ^4^ School of Public Health, Weifang Medical University, Weifang, Shandong, China; ^5^ Shandong Provincial Key Laboratory of Infectious Disease Control and Prevention, Shandong Center for Disease Control and Prevention, Jinan, Shandong, China

**Keywords:** COVID-19, Omicron, booster vaccination, breakthrough infection, scRNA-seq, peripheral immune responses

## Abstract

**Introduction:**

The high percentage of Omicron breakthrough infection in vaccinees is an emerging problem, of which we have a limited understanding of the phenomenon.

**Methods:**

We performed single-cell transcriptome coupled with T-cell/B-cell receptor (TCR/BCR) sequencing in 15 peripheral blood mononuclear cell (PBMC) samples from Omicron infection and naïve with booster vaccination.

**Results:**

We found that after breakthrough infection, multiple cell clusters showed activation of the type I IFN pathway and widespread expression of Interferon-stimulated genes (ISGs); T and B lymphocytes exhibited antiviral and proinflammatory-related differentiation features with pseudo-time trajectories; and large TCR clonal expansions were concentrated in effector CD8 T cells, and clonal expansions of BCRs showed a preference for IGHV3. In addition, myeloid cells in the BA.5.2 breakthrough infection with the fourth dose of aerosolized Ad5-nCoV were characterized by enhanced proliferation, chemotactic migration, and antigen presentation.

**Discussion:**

Collectively, our study informs the comprehensive understandings of immune characterization for Omicron breakthrough infection, revealing the positive antiviral potential induced by booster doses of vaccine and the possible "trained immunity" phenomenon in the fourth dose of aerosolized Ad5-nCoV, providing a basis for the selection of vaccination strategies.

## 1 Introduction

Fo COVID-19 caused by severe acute respiratory syndrome coronavirus 2(SARS-CoV-2) has brought about millions of deaths globally, making it one of the worst pandemics in human history (1). On May 5, 2023, the World Health Organization declared that the COVID-19 pandemic no longer constitutes a Public Health Emergency of International Concern (PHEIC), but it remains a persistent health threat and a complex public health challenge.

The rapid development, emergency approval, and mass vaccination of multiple COVID-19 vaccines were once considered effective means of ending pandemics, but with the emergence of Omicron variants with a high degree of immune evasion and transmission, and the attenuation of vaccine protection due to the dynamics of immune responses, vaccinees may still be infected with Omicron (2, 3). To control the Omicron epidemic, countries around the world have actively implemented a series of vaccination strategies, such as the third booster (4), the fourth booster (5, 6), and the heterologous booster strategy (7, 8), but breakthrough infections are still occurring (9). More importantly, the immune system of breakthrough infection individuals has repeatedly been exposed to different SARS-CoV-2 antigens caused by vaccines and infections, forming a complex “hybrid immunity”. However, we have little knowledge of this complex immune response mechanism and immunoprotection and lack new insights into the immune characteristics of breakthrough-infected individuals.

Single-cell RNA sequencing (scRNA-seq) can conduct unbiased high-throughput studies from the minimum sample size, decipher cell heterogeneity, analyze cell status and function, and comprehensively profile immune responses at single-cell resolution. Since COVID-19, scRNA-seq has been widely used to explore the pathogenesis of viruses, describe the immune characteristics of patients, and screen clinical therapeutic targets (10–13). Recent studies have also explored the immune responses to BNT162b2 (14), Ad5-nCoV (15), and BBIBP-CorV (16) across the entire transcriptome based on single-cell resolution, revealing various individual immunodynamics induced by different vaccines. scRNA-seq has played an important “microscope” role in viral infection and vaccination, providing a large number of meaningful studies for understanding and controlling COVID-19.

To gain a deeper understanding and identify the possible cellular and molecular response mechanisms of Omicron waves in the context of vaccination, we performed scRNA-seq together with single-cell V(D)J sequencing using PBMCs from nine Omicron infections and six naïve with booster vaccination.

## 2 Material and methods

### 2.1 Study design

We recruited nine patients diagnosed with Omicron infection and six vaccinees to participate in this study from October to December 2022. All patients were tested by real-time reverse-transcriptase PCR (RT-PCR) using the SARS-CoV-2 nucleic acid detection kit (Biogerm, China), and the SARS-CoV-2 RNA sequence was confirmed by whole genome sequencing on a Nextseq2000(Illumina, USA). All vaccinees had no history of SARS-CoV-2 infection.

### 2.2 Isolation of PBMCs

The EDTA anticoagulant peripheral blood from patients and vaccinees was collected in the acute phase (within 7 days post symptom onset) and over 1 month after the last vaccination, respectively. The PBMCs of all participants were isolated from anticoagulant venous blood by density gradient sedimentation using Lymphoprep™ density gradients (Axis-Shield, Norway), frozen in cell-saving media, and stored in liquid nitrogen.

### 2.3 Single-cell library preparation and sequencing

#### 2.3.1 Sample preparation

Frozen PBMC samples were thawed quickly in a water bath at 37°C and washed with 5 ml PBS (Gibco, USA) containing 0.04% bovine serum albumin (BSA) (Thermo Fisher Scientific, USA). The Red blood cell lysis solution (Miltenyi Biotec, GER), A 30 µm cell strainer (Miltenyi Biotec, GER), and low-speed centrifugation (200x g for 5 min) were used to remove erythrocytes, clumps, and platelets from the sample. The cell number and viability were determined using an automatic fluorescence counter (Luna, Kr). The concentration of the sample with cell activity greater than 90% was adjusted to 1000 cells/μl in PBS containing 0.04% BSA.

#### 2.3.2 Gel bead-in-emulsion generation and barcoding

Because of 65% cell processing efficiency, aiming for an estimated 10000 cells per library, 16,000 cells per reaction, gel beads and partitioning oil were loaded to Chromium Next GEM Chip K for gel GEM generation and barcoding on Chromium Controller (10X genomics, USA). The GEMs were transferred to prepare GEM-RT for incubation.

#### 2.3.3 Post GEM-RT cleanup and cDNA amplification

After breaking the water-in-oil structure, the post GEM-RT incubation was cleaned up by Dynabeads. Purified cDNA was amplified for 13 cycles and cleaned up using SPRIselect beads (0.6X). The undiluted cDNAs were then run on a Qubit 4.0 Fluorometer (Life Technologies, USA) to determine the cDNA concentration.

#### 2.3.4 5′ Gene Expression and V(D)J library construction

According to the chromium next GEM single cell 5’ v2 user guide, 50 ng cDNA per sample was used to construct 5′ Gene Expression (GEX) library followed by the fragmentation, end repair, A-tailing, adaptor ligation, index PCR, and SPRIselect beads cleaned up. Due to the limited content of V(D)J, V(D)J amplification and clean up from cDNA are required before library construction. 50 ng amplificated cDNA per sample was used to construct the V(D)J library according to the manufacturer’s protocol.

#### 2.3.5 Sequencing

The final library size and quality were evaluated using an Agilent High Sensitivity DNA Kit (Agilent Technologies, CA), and the concentration was checked using the Qubit dsDNA Assay Kit on Qubit 4.0 Fluorometer (Life Technologies, USA). The library was sequenced using an Illumina NextSeq 2000 platform to generate 100 bp paired-end reads. A median sequencing depth of 30,000~50,000 reads/cell was targeted for each 5′ Gene Expression Library and 5000 reads/cell for the V(D)J libraries.

### 2.4 5′ Gene Expression scRNA-seq analysis

CellRanger software (10× Genomics, v.7.0.0) with the default settings was used to align the sequenced fastq files to the human reference version GRCh38 and generate a unique molecular identifier (UMI)–filtered gene and protein expression count matrix for each sample by the “Cellranger count” function. The following quality control steps were performed to filter the count matrices: 1) min. cells = 3; 2) 200 < nFeature_RNA < 6000; 3) percent.mitochondrial<5; 4) percent.erythrocyte < 0.05. For each cell, normalization by “NormalizeData”, highly variable feature calculation by “FindVariableFeatures”, scaling by “ScaleData” and linear dimensionality reduction by “RunPCA” were achieved using the Seurat package (v.4.3.0.1) (17). The Harmony package (v.0.1.1) (18) was used on the integrated object for batch-effect correction. The RunHarmony function was used to integrate variances originating from different data sources and to create harmony embeddings. The “RunUMAP”, “FindNeighbors” and “FindClusters” functions in Seurat were used for Euclidean distance calculations, shared nearest neighbor graph construction, cell clustering, and nonlinear dimensionality reduction. The “FindAllMarkers” function was used to find markers for each cluster. The cell clusters were then preliminarily classified and annotated based on the known markers (10, 19, 20).

### 2.5 Identification of differential expression genes

Differential gene expression testing was performed using the “FindMarkers” function in Seurat with the default parameter “test. use = Wilcox, min. pct = 0.1” and the Benjamini–Hochberg method was used to estimate the adjusted p-value. DEGs were filtered using a 2^^logFC_cutoff^ criteria and a maximum adjusted p-value of 0.05.

### 2.6 Functional and pathway enrichment analysis

The prospective Gene Ontology (GO) terms of DEGs from different groups and cell clusters were identified using clusterProfiler packages (v.4.8.3) (21). The Bonferroni-adjusted P < 0.05 was used as the cut-off criterion.

### 2.7 Definition of cell state scores

For assessing the immune state, the “AddModuleScore” function in Seurat was used to calculate the modular score of pathways with default settings. The following gene sets, response to type I interferon (GO: 0034340), defense response to virus (GO: 0051607), negative regulation of viral process (GO: 0048525), and interferon-mediated signaling pathway (GO:0140888) were used to evaluate each cell score. The gene sets were obtained from AmiGO 2(https://amigo.geneontology.org).

### 2.8 Single-cell trajectories analysis

We used reversed graph embedding to describe the single-cell trajectory of each sample in a fully unsupervised manner by the Monocle package (v.2.28.0) (22). DDRTree was used to learn a principal tree on a population of single cells by default, asserting that it describes the sequence of changes to global gene expression levels as a cell progresses through the biological process under study. We detected genes that followed similar kinetic trends along the CD8 T cell trajectory as well as the B cell trajectory from the starting state. Based on the expression patterns, a hierarchical clustering method was applied to cluster the genes into several subgroups, and the functions of each subgroup were further analyzed by clusterProfiler packages (v.4.8.3).

### 2.9 TCR and BCR analysis

CellRanger software (10× Genomics, v.7.0.0) with the default settings was used to perform V(D)J sequence assembly and paired clonotype calling by the “Cellranger vdj” function. We projected T/B cells with TCR/BCR clonotypes on a umap plot using barcode information by the scRepertoire package (v.1.10.1) (23).

## 3 Results

### 3.1 Single‐cell transcription profiling overview from Omicron breakthrough infection

**Figure 1:**
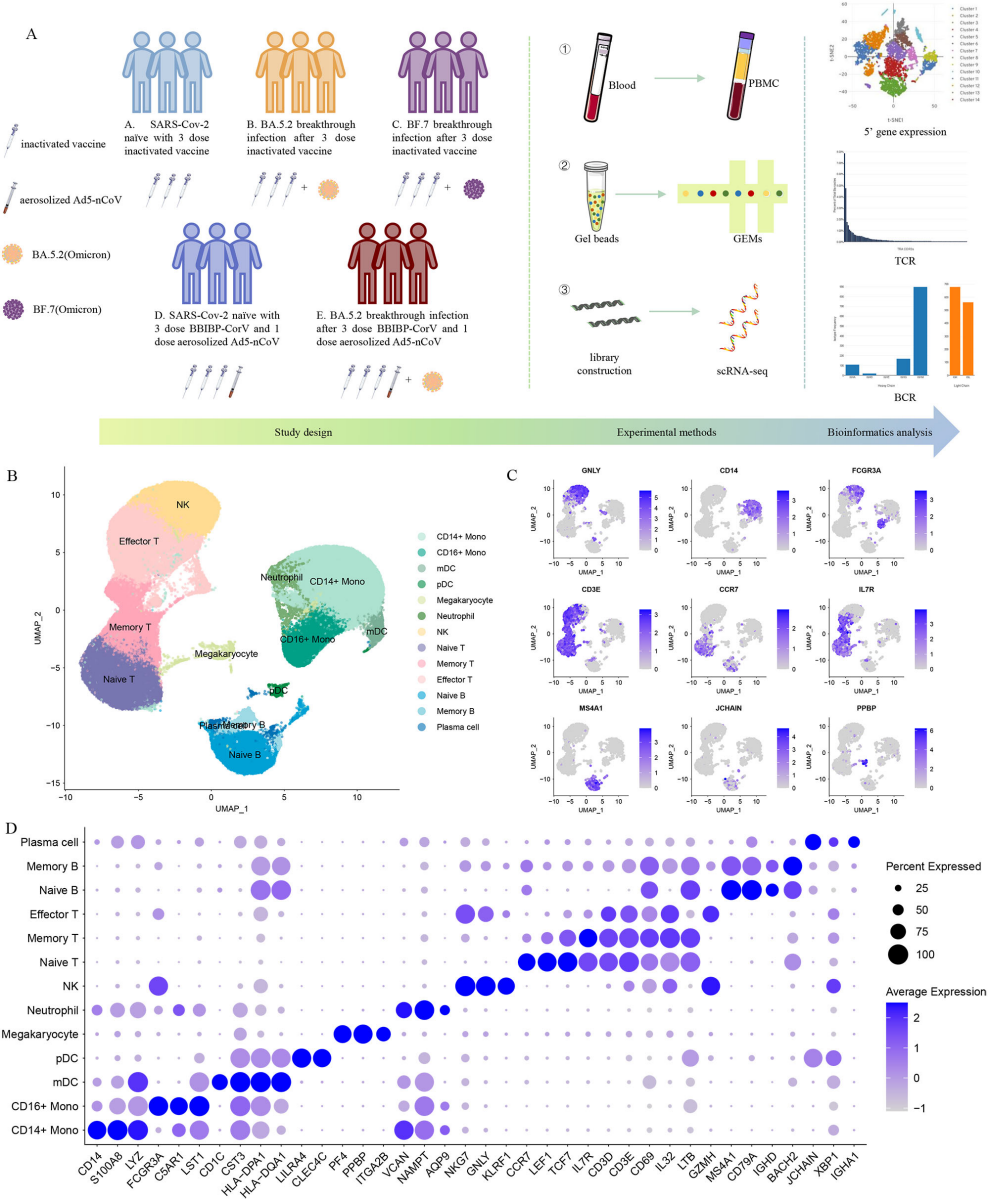
Single-cell RNA sequencing of peripheral blood cells from Omicron breakthrough infection. **(A)** Overview of the study, including the details of study design and overall experimental. **(B)** UMAP projections of all 123,531 high-quality single cells derived from patients with Omicron breakthrough infection and naïve with booster vaccination. Cells are colored by cell type identity. Each dot represents a single cell. **(C)** Normalized expression levels of the marker genes that are used to classify clusters as represented in the UMAP plot. **(D)** Dot plots of 5’ gene expression where the color is scaled by average expression and the dot size is the percent of the expressed cells of marker genes in each labeled cell type.

To characterize the peripheral immune responses to Omicron breakthrough infection, we recruited nine laboratory-confirmed Omicron infections and six naïve with booster vaccination to perform the droplet‐based scRNA‐seq. Their ages ranged from 23 to 57 years old, and 7 of them were male. All participants were split into five groups with distinct antigen exposure combinations: group A (SARS-CoV-2 naïve with the third dose of inactivated vaccine), group B (BA.5.2 breakthrough infection after the third dose of inactivated vaccine), group C (BF.7 breakthrough infection after the third dose of inactivated vaccine), group D (SARS-CoV-2 naïve with three inactivated vaccine doses and the fourth dose of aerosolized Ad5-nCoV) and group E (BA.5.2 breakthrough infection after three inactivated vaccine doses and the fourth dose of aerosolized Ad5-nCoV) (**Figure 1A**). All the inactivated vaccines mentioned above are BBIBP-CorV inactivated vaccines, which are the first generation of inactivated vaccines against wild-type SARS-CoV-2, and adenovirus vector vaccines Ad5-nCoV are also the first generation of vaccines.

We sequenced 153,395 cells from 15 samples (**Supplementary Figure S1A**). Following stringent quality-control criteria, a total of 123,531 high-quality single cells were ultimately obtained(**Supplementary Figure S1B**). We identified 13 major cell clusters with distinct transcriptomic signatures, including CD14^+^ monocytes (*CD14, S100A8, LYZ*), CD16^+^ monocytes (*FCGR3A, C5AR1, LST1*), mDCs (*CD1C, CST3, HLA-DPA1*), pDCs (*LILRA4, CLEC4C*), megakaryocytes (*PF4, PPBP, ITGA2B*), neutrophils (*VCAN, NAMPT, AQP9*), natural killer (NK) cells (*NKG7, GNLY, KLRF1*), naive T cells (*CCR7, IL7R, LEF1*), memory T cells (*IL7R, CD3D, CD3E*), effector T cells (*GZMH, CD3D, CD3E*), naive B cells (*MS4A1, CD79A, IGHD*), memory B cells (*MS4A1, CD79A, BACH2*), plasma cells (*JCHAIN, IGHA1, XBP1*) (**Figures 1B–D**).

**Figure 2:**
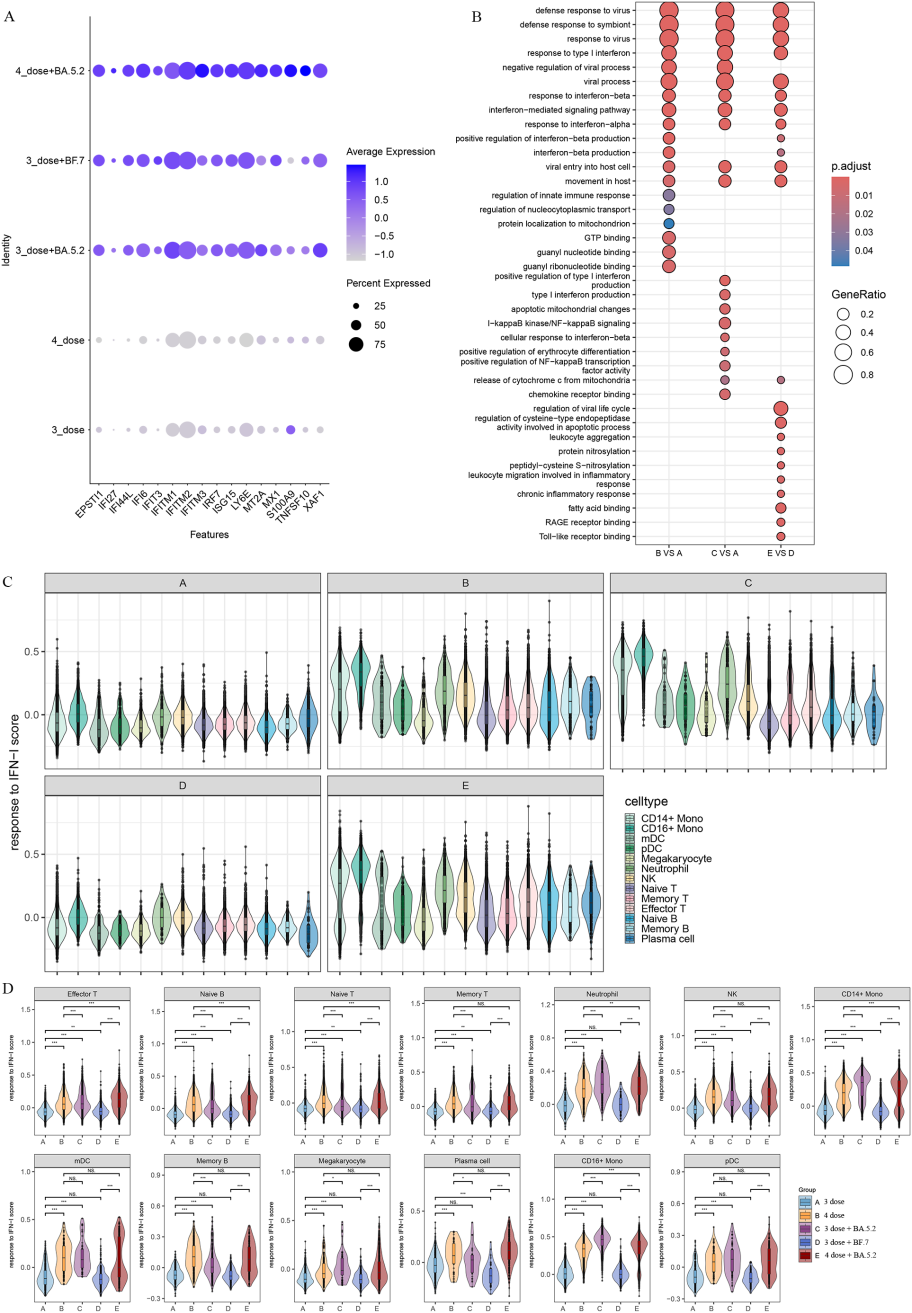
Patients with Omicron breakthrough infection have gene expression profiles indicative of higher interferon (IFN) response. **(A)** Average expression of differentially expressed genes (DEGs) from Omicron breakthrough infection groups. The color is scaled by average expression and the dot size is the percent of the expressed cells of DEGs. **(B)** GO enrichment analysis showing the terms of the up-regulated DEGs between patients with Omicron breakthrough infection and naïve with booster vaccination under the same vaccination background. **(C)** Violin plot of response to type I IFN module genes for each cell from patients with Omicron breakthrough infection and naïve with booster vaccination. The color indicates each labeled cell type. **(D)** Violin plots of response to type I IFN module genes of each group split by cell types. The color indicates each group. Wilcoxon test was used for pairwise comparisons, “***”, p <0.001, “**”, p <0.01, “*”, p <0.05, and “NS” indicates no statistical significance.

To analyze the biological significance of single-cell transcriptional changes in the function of Omicron breakthrough infection, we performed differential expression gene (DEGs) and pathway enrichment analysis between Omicron breakthrough infection and naïve groups in the same vaccination context. We found some upregulated DEGs after Omicron breakthrough infection, including lymphocyte antigen 6E(*LY6E*) involved in T-cell development, *S100A9* regulation of inflammatory processes and immune response, tumor necrosis factor ligand superfamily member 10(*TNFSF10*), XIAP-associated factor 1(*XAF1*) induced apoptosis and multiple interferon stimulated genes (including *IFI6, IFI27, IFI44L, IFIT3, IRF7, ISG15*, and *IFITM1/2/3*) (**Figure 2A**). Gene Ontology (GO) enrichment analysis revealed abundant immune system processes, particularly the type I interferon (IFN-I) pathway (**Figure 2B**). The IFN-I response was further assessed by using the “response to IFN-I” module score, which revealed elevated scores for most cell clusters in Omicron breakthrough infection(**Figure 2C**). Notably, patients with the fourth dose of aerosolized Ad5-nCoV had even higher scores for CD14^+^ monocytes, CD16^+^ monocytes, neutrophils, naive T cells, effector T cells, and naive B cells (**Figure 2D**).

### 3.2 Transcriptomic differences of myeloid cells after Omicron breakthrough infection

**Figure 3:**
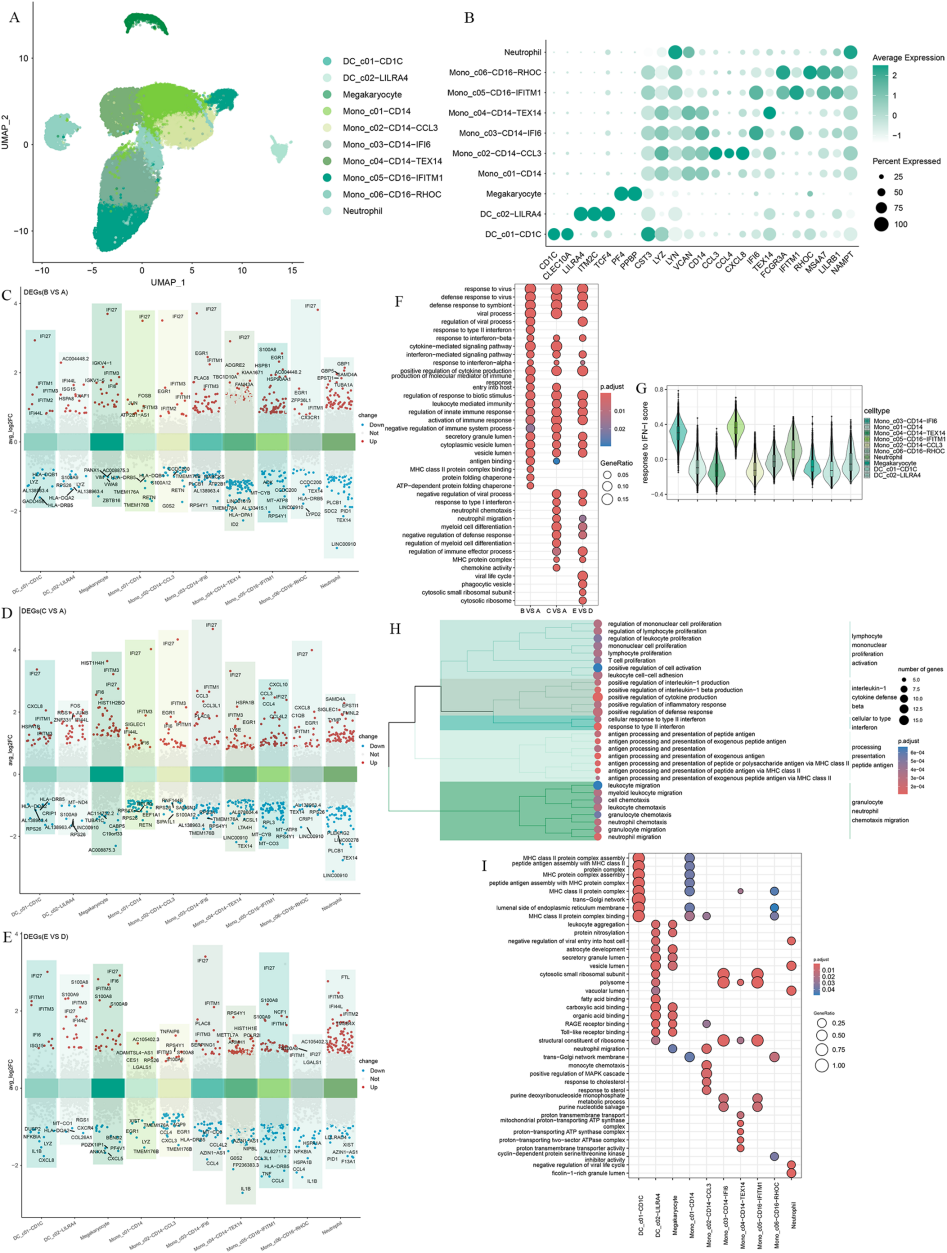
Differential gene expression and transcriptional profiling functional analysis in myeloid cells after Omicron breakthrough infection. **(A)** UMAP visualization of myeloid cells identified from gene expression data. Cells are colored by cell type identity. Each dot represents a single cell. **(B)** Dot plots of marker gene expression for myeloid cell subtypes. **(C)** Volcano plots of DEGs between group B(BA.5.2 breakthrough infection after the third dose of inactivated vaccine) and group A(SARS-Cov-2 naïve with the third dose of inactivated vaccine) for myeloid cell subtypes. **(D)** Volcano plots of DEGs between group C(BF.7 breakthrough infection after the third dose of inactivated vaccine) and group A(SARS-Cov-2 naïve with the third dose of inactivated vaccine) for myeloid cell subtypes. **(E)** Volcano plots of DEGs between group E(BA.5.2 breakthrough infection after three BBIBP-CorV doses and the fourth dose of aerosolized Ad5-nCoV) and group D(SARS-Cov-2 naïve with three BBIBP-CorV doses and the fourth dose of aerosolized Ad5-nCoV) for myeloid cell subtypes. **(F)** GO enrichment analysis showing the terms of the up-regulated DEGs from myeloid cells between patients with Omicron breakthrough infection and naïve with booster vaccination under the same vaccination background. **(G)** Violin plot of response to type I IFN module genes for each cell from all participants. The color indicates each labeled myeloid cell type. **(H)** Functional enrichment cluster analysis of up-regulated DEGs between group E(BA.5.2 breakthrough infection after three BBIBP-CorV doses and the fourth dose of aerosolized Ad5-nCoV) and group B(BA.5.2 breakthrough infection after the third dose of inactivated vaccine). **(I)** GO enrichment analysis showing the terms of the up-regulated DEGs from each myeloid cell type between group E(BA.5.2 breakthrough infection after three BBIBP-CorV doses and the fourth dose of aerosolized Ad5-nCoV) and group B(BA.5.2 breakthrough infection after the third dose of inactivated vaccine).

To further characterize myeloid cells, we clustered the myeloid immune cells into 10 subsets, including two dendritic cell subsets(DC_c01-CD1C, DC_c02-LILRA4), four CD14^+^ monocyte subsets(Mono_c01-CD14, Mono_c02-CD14-CCL3, Mono_c03-CD14-IFI6, Mono_c04-CD14-TEX14), two CD16^+^ monocyte subsets(Mono_c05-CD16-IFITM1, Mono_c06-CD16-RHOC), a megakaryocyte and neutrophil subsets (**Figures 3A, B**).

We compared the gene expression profiles of each cell cluster between infected and vaccinated individuals and found that interferon alpha inducible protein 27 (*IFI27*) showed significant upregulation in the breakthrough infection group, especially in Mono_c03-CD14-IFI6 with interferon-related characteristics (**Figures 3C–E**). As expected, the “response to virus”, “defense response to virus”, interferon and cytokine-mediated signaling pathways, and the activation of immune response were universally enriched(**Figure 3F**), indicating the effective response of myeloid cells to breakthrough infection. We noticed a certain degree of heterogeneity in the GO enrichment analysis of the three breakthrough infection groups compared to the vaccination group. In the breakthrough infection after the third dose of inactivated vaccine, BA.5.2 infection induced the unique “response to type II interference” and “MHC class II binding”, while BF.7 was more inclined toward “neutral migration” and “myoid cell differentiation”(**Figure 3F**). In peripheral blood, Mono_c03-CD14-IFI6 and Mono_c05-CD16-IFITM1 expressed the highest amount of type I IFN response genes compared to all myeloid cells (**Figure 3G**). To describe the potential role of the fourth dose of aerosolized Ad5-nCoV, we focused on group B and group E for further analysis. Functional enrichment cluster analysis showed that, compared to group B, the upregulated DEGs in group E were enriched through pathways such as “lymphocyte mononuclear promotion activation”, “interleukin-1 cytotoxic defense beta”, “cellular to interference”, “processing presentation peptide antigen”, and “granulocyte neutral chemotaxis migration” (**Figure 3H**). Specifically, DC_ C01-CD1C and Mono_ C01-CD14 performed MHC protein-related antigen presentation functions (**Figure 3I**).

### 3.3 T and NK cell heterogeneity in responding to Omicron breakthrough infection

**Figure 4:**
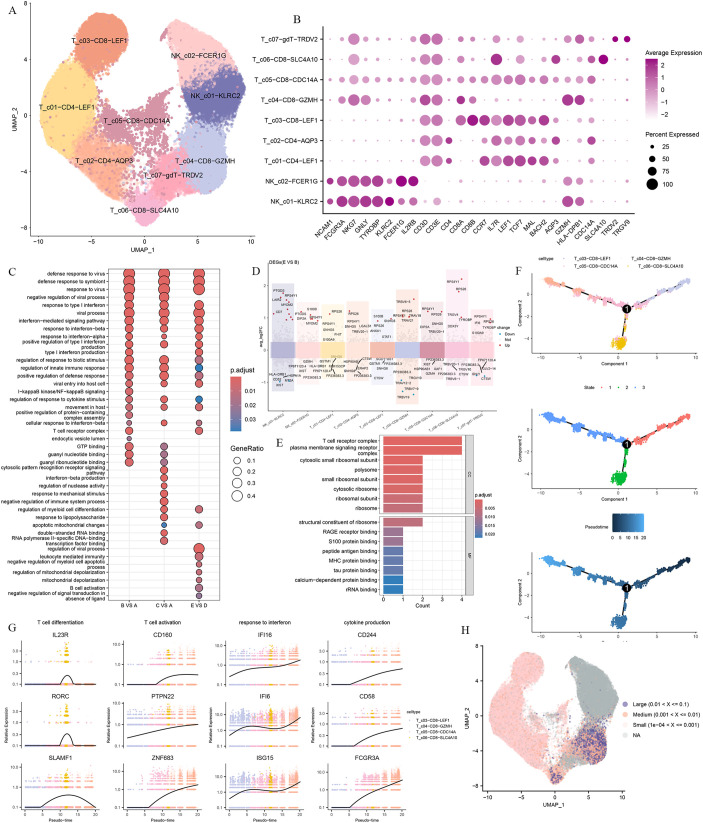
T and NK cells heterogeneity in responding to Omicron breakthrough infection. **(A)** UMAP visualization of T and NK cells identified from gene expression data. Cells are colored by cell type identity. Each dot represents a single cell. **(B)** Dot plots of marker gene expression for T and NK cell subtypes. **(C)** GO enrichment analysis showing the terms of the up-regulated DEGs from T and NK cells between patients with Omicron breakthrough infection and naïve with booster vaccination under the same vaccination background. **(D)** Volcano plots of top DEGs between group E(BA.5.2 breakthrough infection after three BBIBP-CorV doses and the fourth dose of aerosolized Ad5-nCoV) and group B(SARS-Cov-2 naïve with three BBIBP-CorV doses and the fourth dose of aerosolized Ad5-nCoV) for T and NK cell subtypes. **(E)** Functional enrichment analyses in cell component and molecular function of the up-regulated DEGs between group E(BA.5.2 breakthrough infection after three BBIBP-CorV doses and the fourth dose of aerosolized Ad5-nCoV) and group B(SARS-Cov-2 naïve with three BBIBP-CorV doses and the fourth dose of aerosolized Ad5-nCoV). **(F)** Pseudotime trajectories for CD8 T cells based on Monocle2, points are colored by cell type identity, state, and pseudotime. **(G)** Genes expression involved in the function and response of T cells modeled along with the pseudotime of CD8 T cell lineages. **(H)** UMAP projection of the TCR clonetype from patients with Omicron breakthrough infection and naïve with booster vaccination.

To explore the immune characteristics of T cells and NK cells, T cells and NK cells were subclustered into 12 clusters based on the distribution and expression of classical cellular markers. These included two clusters of NK cells (*NKG7, GNLY, TYROBP*), two clusters of CD4 T cells (*CD3D, CD3E, CD4*), four clusters of CD8 T cells (*CD3D, CD3E, CD8A, CD8B*), and a γ-delta T cells cluster (*TRDV2, TRGV9*) (**Figures 4A, B**).

We performed the DGE analysis comparing different groups in each T and NK cell cluster and identified genes associated with “response to type I interferon”, “regulation of response to cytotoxic stimuli”, and “T cell receptor complex” that were activated after infection. As with myeloid cells, we also observed heterogeneity involving immune signaling pathways in the infected group with distinct antigen exposure combinations. Functional enrichment analysis showed that upregulated DEGs in group E VS group D were specifically enriched in “leukocyte mediated immunity”, “negative regulation of myeloid cell apoptotic process” and “B cell activation” pathways (**Figure 4C**). In summary, this indicates that there is a coordinated T cell and B cell response during the BA.5.2 breakthrough infection with the fourth dose of aerosolized Ad5-nCoV, resulting in coupled antiviral adaptive immunity. The DEG analysis showed that *LAIR2, CD7, KLRB1, TYROBP, TRAV19, TRAV21*, and *TRBV6-5* were upregulated in group E VS group B both breakthrough infection by BA.5.2 (**Figure 4D**). These DEGs were enriched in the “T cell receptor complex”, “peptide antigen binding” and “MHC protein binding” pathways (**Figure 4E**).

For CD8 T cells, we defined a naive CD8 T cell cluster (T_c03-CD8-LEF1), a memory CD8 T cell cluster(T_c05-CD8-CDC14A), and two effector CD8 T cell clusters(T_c04-CD8-GZMH, T_c06-CD8-SLC4A10). The lineage relationships between CD8 T subsets were inferred and presented in a tree structure diagram. The trajectory constitutes one decision point and three states, which indicates that naive CD8 T cells are connected directly with memory CD8 T cells followed by effector CD8 T cells (**Figure 4F**). The trend of the T cell differentiation first increased and then decreased with the pseudo-time. Along with this trend, the T cell activation, response to interferon, and cytokine production gradually increased (**Figure 4G**).

**Figure 5:**
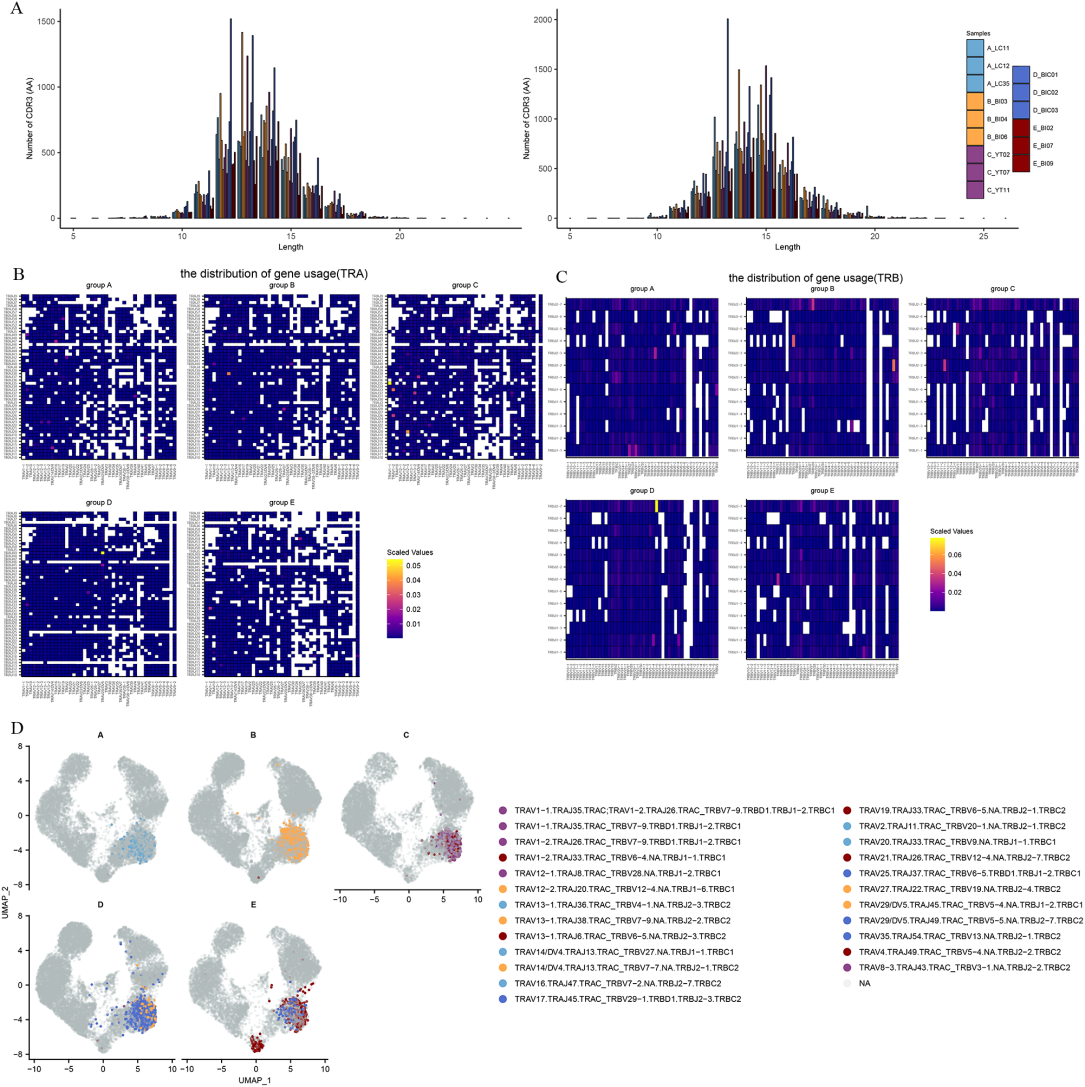
The global profile of expansion and specific rearrangements of TCR V/J. **(A)** CDR3 distribution of T cell clones from TRA (left) and TRB (right). **(B)** Heatmap showing the distribution of V/J gene usage in TRA. **(C)** Heatmap showing the distribution of V/J gene usage in TRB. **(D)** UMAP projection of the top five expanded clones from patients with Omicron breakthrough infection and naïve with booster vaccination.

To investigate the TCR repertoire dynamics during immune responses to Omicron, we analyzed single-cell TCR sequencing data. The UMAP visualization of these TCR clones showed that the larger clone mainly expanded in the effector CD8 T cells (**Figure 4H**). This suggests that the amplified effector CD8 T cell clone plays a critical role in fighting against the virus. Then we assessed CDR3 length across the TCR α and β CDR3 sequences from the five groups. The TCR α chain CDR3 length floated mainly between 7 and 20 aa. The TCR β chain CDR3 length floated between 9 and 22 aa (**Figure 5A**). Then we evaluated the distribution of the V/J gene of the TCR in all participants. Our data showed a high level of *TRAV1-2, TRAV3-1, TRAV29/DV5, TRAV21, TRAJ33, TRAJ20*, and *TRAJ49* expression in the TCR α chain construction (**Supplementary Figures S2A, B**). Moreover, all groups showed the top use of *TRBV20-1, TRBJ2-1*, and *TRBJ2-7* in the TCR β chain construction (**Supplementary Figures S2C, D**).

V–J pairing distribution analysis was used to display the different V–J combinations. We found that TRAV16/TRAJ47, TRAV13−1/TRAJ38, TRAV1−1/TRAJ35, TRAV29/DV5/TRAJ49 and TRBV13/TRBJ2-1 were more frequently used in groups A-E. It is worth noting that TRAV1-2/TRAJ33 showed an increase in scaled values in all five groups, suggesting that this may be a specific dominant connection mediated by both vaccines and infections (**Figure 5B**). In the TCR β chain, TRBV5−1/TRBJ2−3, TRBV19/TRBJ2-4, TRBV11−3/TRBJ2−2, TRBV5−5/TRBJ2−7, and TRBV13/TRBJ2−1 dominant connections were observed in the five groups respectively (**Figure 5C**). The top five TCR clones were highlighted in the different enriched cell clusters in different groups (**Figure 5D**). We found that CD8 effector T cells (T_c04-CD8-GZMH) in the BA.5.2 breakout infection with the fourth aerosolized Ad5-nCoV shared some of the same TCR clones and rearrangements in the SARS-CoV-2 naïve with the fourth aerosolized Ad5-nCoV, and T_c06-CD8-SLC4A10 also showed abundant TCR clone types only in the BA.5.2 breakout infection with the fourth aerosolized Ad5-nCoV. These continued expansions and specific rearrangements of TCR V(D)J genes may be a potential form of immune memory induced by fourth aerosolized Ad5-nCoV.

### 3.4 B cell remodeling and BCR changes

**Figure 6:**
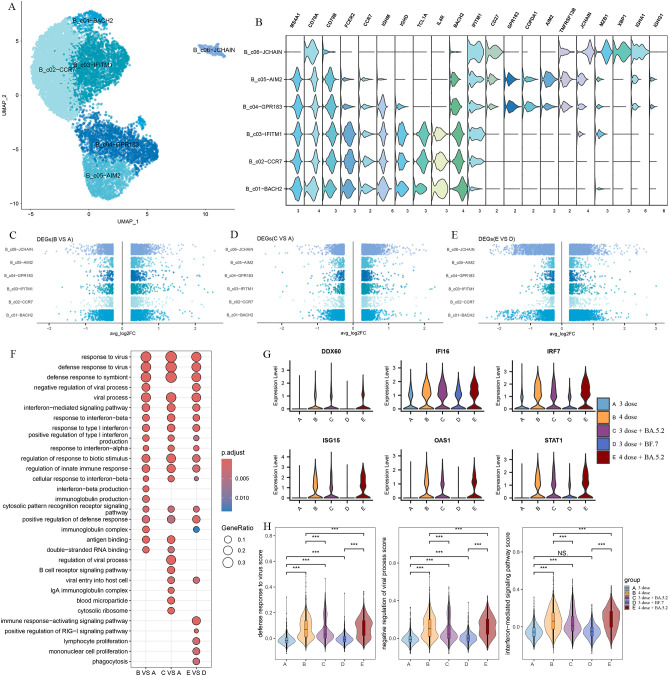
B cell remodeling after Omicron breakthrough infection. **(A)** UMAP visualization of B cells identified from gene expression data. Cells are colored by cell type identity. Each dot represents a single cell. **(B)** Violin plot showing the expression of signature genes in the 6 cell types. **(C)** Volcano plots of DEGs between group B(BA.5.2 breakthrough infection after the third dose of inactivated vaccine) and group A(SARS-Cov-2 naïve with the third dose of inactivated vaccine) for B cell subtypes. **(D)** Volcano plots of DEGs between group C(BF.7 breakthrough infection after the third dose of inactivated vaccine) and group A(SARS-Cov-2 naïve with the third dose of inactivated vaccine) for B cell subtypes. **(E)** Volcano plots of DEGs between group E(BA.5.2 breakthrough infection after three BBIBP-CorV doses and the fourth dose of aerosolized Ad5-nCoV) and group D(SARS-Cov-2 naïve with three BBIBP-CorV doses and the fourth dose of aerosolized Ad5-nCoV) for B cell subtypes. **(F)** GO enrichment analysis showing the terms of the up-regulated DEGs from B cells between patients with Omicron breakthrough infection and naïve with booster vaccination under the same vaccination background. **(G)** Violin plots of selected DEGs involved in response to type I interferon. **(H)** Violin plot of the score of “defense response to virus”, “negative regulation of viral process” and “interferon−mediated signaling pathway” module genes for each group. Wilcoxon test was used for pairwise comparisons, ***, p <0.001 and “NS” indicates no statistical significance.

According to canonical marker expression, B cells and plasma cells were identified into six clusters including three naive B cell clusters(B_c01-BACH2, B_c02-CCR7, B_c03-IFITM1), two memory B cell clusters(B_c04-GPR183, B_c05-AIM2), and a plasma cell cluster(B_c06-JCHAIN) (**Figures 6A, B**). To further investigate the important role in the antiviral response of B/plasma cells, we assessed differential transcriptomic changes in B/plasma cells. In comparison with group A and group D, GO analysis showed that the upregulated DEGs in the Omicron breakthrough infection group were enriched in the “response to virus”, “response to type I interferon”, “cytosolic pattern recognition receptor signaling pathway” and “immunoglobulin complex” pathways (**Figures 6C–F**). We found that the type I interferon (IFN-I)-related genes, *DDX60, IFI16, IRF7, ISG15, OAS1*, and *STAT1*, were more highly expressed after Omicron breakthrough infection (**Figure 6G**). Meanwhile, BA.5.2 breakthrough infection with the fourth dose of aerosolized Ad5-nCoV expressed the highest amount of “defense response to virus”, “negative regulation of viral process” and “interferon−mediated signaling pathway” genes compared to other groups (**Figure 6H**).

**Figure 7:**
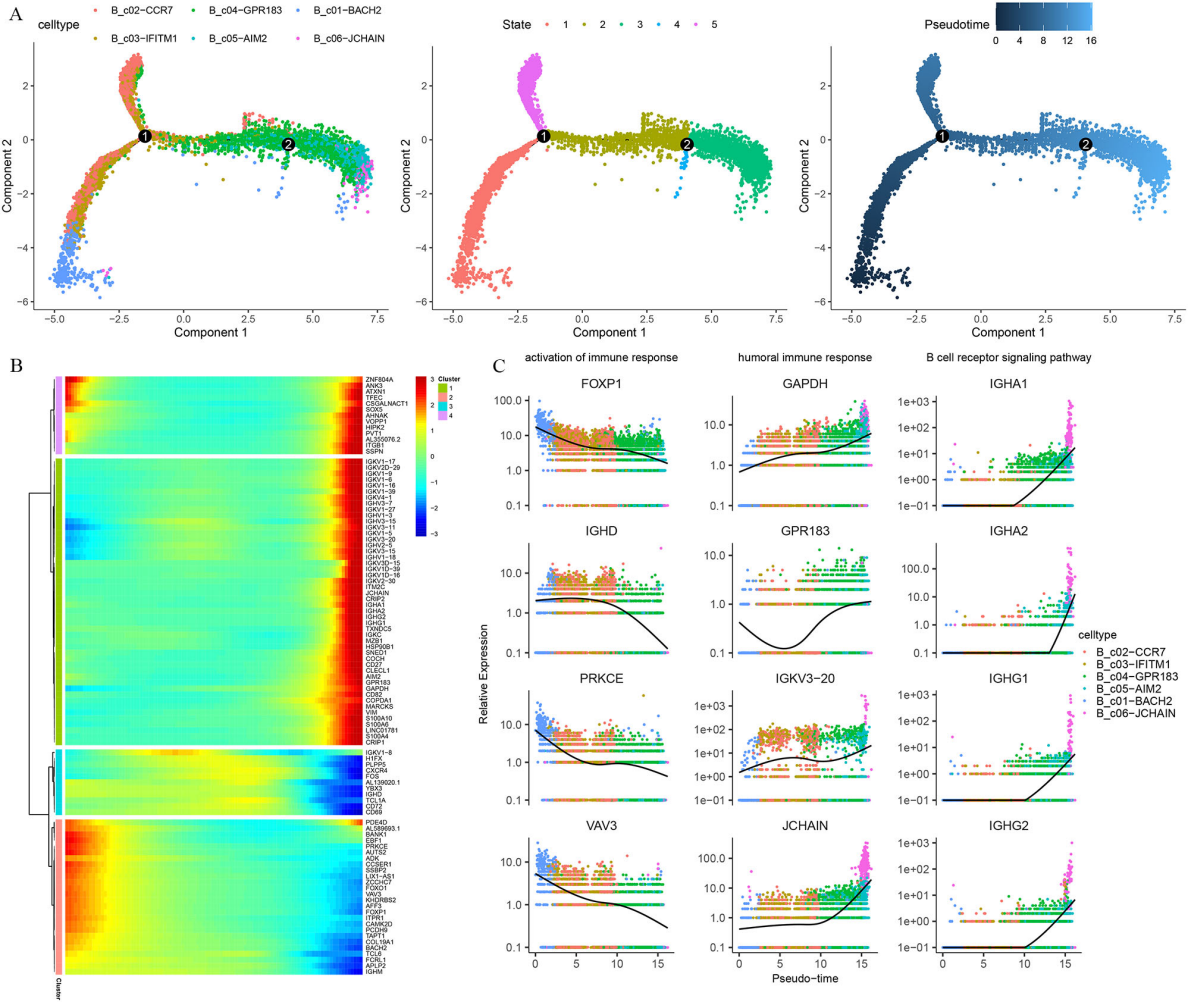
Pseudo-time trajectory reconstruction with B cells subpopulation. **(A)** Pseudotime trajectories for B cells based on Monocle2, points are colored by cell type identity, state, and pseudotime. **(B)** Gene expression dynamics along the B cells lineage. The top 100 genes were clustered into 4 gene sets, and each of them was characterized by specific expression profiles. **(C)** Genes expression involved in the function and response of B cells modeled along with the pseudotime of B cell lineages.

Trajectory analysis was used to describe dynamic changes in gene expression behind each B-cell subtype in a more detailed way. Pseudo-time trajectory analysis showed that the arrangement of cells on the pseudotime line was naive B cells and memory B cells to plasma cells, and cells in state 2 were mainly memory B cells (**Figure 7A**). Unsupervised analysis divided the top 100 genes into 4 sets. Gene sets 2 and 3 were enriched with genes expressed early and middle in the trajectory, while gene sets 1 and 4 were enriched in the late of trajectory(**Figure 7B**). To further explore the functional enrichment of the cell states, GO analysis revealed that the trend of the activation of the immune response decreased with the pseudo-time and the increase in the humoral immune response, B cell receptor signaling pathway (**Figure 7C**).

To understand the dynamics of the BCR repertoire during immune responses to Omicron breakthrough infection, we reconstructed BCR sequences by scBCR-seq. We found the skewing of *IGHV3-23, IGHV3-33, IGHJ4, IGKV1−39, IGKV3−20* and *IGLJ2* in BCR chain construction (**Supplementary Figures S3A–D**). To further investigate the V-J rearrangement characteristics of BCR, we analyzed the V-J connections of five groups. The results indicate that the expression of IGHV3-23/IGHJ4 and IGKV2-30/IGKJ5 were increased in group A, IGHV3-23/IGHJ4, and IGLV1−44/IGLJ3 were more frequently observed in group B, the expression of IGHV3-23/IGHJ4 and IGKV1−39/IGKJ2 were increased in the group C, IGHV3-23/IGHJ4 and IGKV1−39/IGLJ2 were more frequently observed in group D, IGHV3−33/IGHJ3, IGHV3-23/IGHJ4 and IGLV1−51/IGLJ3 were more frequently observed in group E(**Supplementary Figures S4A, B**). We next examined biases and perturbations of the top 10 clones in IGH and IGL by comparing repertoire assignments across the five groups (**Supplementary Figures S4C, D**). Taken together, our results show that BCR V/J is used at similar frequencies in the vaccinees and breakthrough infection groups, showing germline preference. In addition, expanded clonotypes were found across all major cell types with larger clonotypes present primarily in plasma cell clusters (**Supplementary Figure S4E**).

## 4 Discussion

Establishing immune protection against SARS-CoV-2 through safe and effective vaccination has been considered a key measure to reduce COVID-19 transmission and health damage, but now the breakthrough infection of Omicron among vaccine recipients has become an emerging issue and has brought limited awareness. We systematically profiled the response and immune characteristics of the peripheral immune to Omicron (BA.5.2 and BF.7) breakthrough infection under different vaccine antigen exposure backgrounds using scRNA-seq.

The recognition of viral infection by innate immune sensors activates the type I IFN response, which is the main first line of defense against the virus (24) IFN-I exhibits antiviral function by inducing transcription of interferon-stimulated genes (ISGs), which limits a series of processes of viral replication (25). In this study, all patients with Omicron breakthrough infection showed significantly enhanced interferon-mediated antiviral profiles during the acute phase. The “response to type I interferon”, “response to interferon-beta”, “interferon-mediated signaling pathway” and “response to interferon-alpha” pathways were enriched in most cell clusters, as well as high expression of ISGs (*IFI6, IFI27, IFI44L, IFIT3, IRF7, and ISG15*), compared with the vaccination group. According to the score of the interferon gene set, monocytes have a higher IFN-I score. In further analysis of myeloid cells, CD14^+^monocyte (Mono_c03-CD14-IFI6) and CD16^+^monocyte (Mono_c05-CD16-IFITM1) with IFN related characteristics were found, demonstrating a more pronounced IFN-I response. The ability of SARS-CoV-2 to downregulate the host IFN-I response has been demonstrated as a viral strategy for evading host immunity (26, 27). Previous studies have found that some patients with severe COVID-19 experience impaired and delayed interferon responses, as well as downregulation of ISGs (28, 29). Peripheral blood monocytes have a deficiency in their response to IFN signaling and may be exhibit immunoparalysis, leading to a kind of immune dysregulation (30). In our study, there was no such selective dysregulation with a depressed IFN response, which may be one of the important reasons for the reduction in severe disease after breakthrough infection.

Adaptive T-cell immunity and B-cell immunity are of great significance for the successful clearance of viruses. We found that the “response to type I interferon”, “regulation of response to cytokine stimulus” and “T cell receptor complex” pathways were enriched and activated in T cell clusters after breakthrough infection. During trajectory analysis of CD8 T cell subtypes, differentiation, activation, and pro-inflammatory characteristics of T cells were observed. The high expression of granzyme genes in effector CD8 T cells (T_c04-CD8-GZMH) in the terminal differentiation state is one of the important cellular immune pathways that induces apoptosis in virus-infected cells. TCR analysis confirmed that the larger clone mainly expanded in the effector CD8 T cells. Together, these interesting findings suggest that effector CD8 T cells play an important antiviral role in Omicron breakthrough infection. Previous studies have also found that in SARS-CoV-2 infections, the presence of virus-specific CD8 T cells has been associated with better COVID-19 outcomes (31, 32). For humoral immunity, the “response to type I interferon”, “antigen binding”, “cytosolic pattern recognition receptor signaling pathway” and “immunoglobulin complex” pathways were widely enriched in B cell clusters. Pseudo-time trajectory analysis showed that B cell clustering differentiated from naïve B cells involved in immune response activation functions, to memory B cells and plasma cells that undertake humoral immunity and B cell receptor signaling pathways. In addition, previous studies have shown that patients with COVID-19 prioritize the usage of *IGHV3* subfamily genes, mostly rearranged with *IGHJ4* and *IGHJ6* (33, 34), and asymptomatic patients have a higher proportion of using *IGHV3* and *IGHJ4* (35). We also found high-frequency usage and rearrangement of the IGHV3-33-IGHJ4 and IGHV3-23-IGHJ4 fragments. Taken together, these findings help to illustrate the underlying molecular basis of breakthrough infection, leading to a better understanding of the mechanisms of adaptive immune response.

Following the adjustment of prevention and control policies in China before December 2022, the BA.5.2 and BF.7 variants triggered the first Omicron wave (36). They both originated from the BA.5 lineage of the Omicron variant, but BF.7 has an additional R346T amino acid mutation site on the Spike(S) protein compared to BA.5.2. Mutations in the S protein, which largely affect conformation and neutralizing antibody epitopes of the virus, have been directly linked to infectivity, transmissibility, and immune evasion capabilities (37). Several studies have found a certain degree of difference in the neutralizing ability of serum from vaccinations and breakthrough infections to BA.5.2 and BF.7 (38, 39). Previous studies have also found a higher incidence of hyperthermia in patients infected with BA.5.2 than those infected with BF.7 (40). Now, we have analyzed the immune response characteristics induced by BA.5.2 and BF.7 breakthrough infection with the third dose of inactivated vaccine at the single-cell transcription level. Overall, they induced partially identical host antiviral responses, including “defense response to virus”, “response to type I interferon” and “negative regulation of viral process”, but with their distinctive features. For example, BA.5.2 breakthrough infection induced “T cell receptor complex” in T cell clustering, while in B cell clustering, BF.7 breakthrough infection induced an “IgA immunoglobulin complex” pathway that was not present in those infected with BA.5.2. These transcriptional profile distinctions may be potential mechanisms to explain differences in neutralization capacity and clinical features.

We focused on group B and group E which were both infected by BA.5.2 for further analysis, attempting to compare the potential effects of different vaccination strategies. We found that cells contributed by group E showed higher “response to IFN-I”, “defense response to virus”, “negative regulation of virtual process”, and “interference mediated signaling pathway” scores. The “lymphocyte mononuclear proliferation activation”, “interleukin-1 cytokine defense beta”, “cellular to interferon”, “processing presentation peptide antigen” and “granulocyte neutrophil chemotaxis migration” performed by myeloid cells were significantly enriched. A growing body of literature indicates that innate immune cells can also exhibit adaptive characteristics after certain infections or vaccines, which are functionally similar to building immune memory, called trained immunity (41–43). Previous studies have found that BNT162b2 (14) and ChAdOx1 nCoV-19 (44) can induce prolonged innate immune activation, characterized by increased defense and pro-inflammatory gene transcription centered on heterogeneous myeloid cell populations. The myeloid cells of the Omicron breakthrough infection with the fourth dose showed enhanced antigen presentation function enrichment entries at the transcriptional level, which may also be related to training immunity, but this needs to be confirmed by further epigenetic research such as functional testing or protein level verification.

Finally, we systematically reviewed existing single-cell transcriptome studies on breakthrough infections. Consistent with the previous three studies (45–47), we mostly observed the antiviral activity, large TCR clonal expansions which concentrated in effector CD8 T cells, and clonal expansions of BCRs. These immune responses are likely to be common peripheral immune features of early Omicron variant strains breakthrough infection after inactivated COVID-19 vaccine and Adenovirus type 5 (Ad5)-vectored COVID-19 vaccine. In addition, the most obvious novelty of this study is that we have taken the lead in exploring breakthroughs infections after three inactivated vaccine doses and the fourth dose of aerosolized Ad5-nCoV, and revealing the positive antiviral potential induced by booster doses of vaccine and the possible “trained immunity” phenomenon in the fourth dose of aerosolized Ad5-nCoV at the transcriptional level.

In summary, our research provides new insights into the cellular and molecular basis of peripheral immune features in Omicron breakthrough infection at single-cell resolution, which has important guiding significance for the selection of vaccination strategies. However, the most significant limitations of this study were the small sample size and the absence of a follow-up study. Further research involving more samples and longer follow-up times is needed to comprehensively understand the longitudinal innate and adaptive immune responses, evaluate the possibility of reinfection, and guide vaccination strategies.

## Data Availability

The datasets presented in this study can be found in online repositories. The names of the repository/repositories and accession number(s) can be found below: GSE248556 (GEO).

## Supplementary material

Supplementary Figure 1 Basic characteristics of the integrated dataset. **(A)** The count, feature, mitochondria percentage, and red blood cell percentage characteristics of 153,395 cells from 15 samples have been sequenced. **(B)** The count, feature, mitochondria percentage, and red blood cell percentage characteristics of 123,531 cells after QC

Supplementary Figure 2 The distribution of V/J gene in TCR. **(A)** The histogram of the distribution of the V gene in TRA across five groups. **(B)** The histogram of the distribution of the J gene in TRA across five groups. **(C)** The histogram of the distribution of the V gene in TRB across five groups. **(D)** The histogram of the distribution of the J gene in TRB across five groups.

Supplementary Figure 3 The distribution of V/J gene in BCR. **(A)** The histogram of the distribution of the V gene in IGH across five groups. **(B)** The histogram of the distribution of the J gene in IGH across five groups. **(C)** The histogram of the distribution of the V gene in IGL/IGK across five groups. **(D)** The histogram of the distribution of the J gene in IGL/IGK across five groups.

Supplementary Figure 4 The profile of expansion and specific rearrangements of BCR V/J. **(A)** Heatmap showing the distribution of V/J gene usage in IGH. **(B)** Heatmap showing the distribution of V/J gene usage in IGL/IGK. **(C)** The biases and perturbations of the top 10 clones in IGH from patients with Omicron breakthrough infection and naïve with booster vaccination. **(D)** The biases and perturbations of the top 10 clones in IGL/IGK from patients with Omicron breakthrough infection and naïve with booster vaccination. **(E)** UMAP projection of the BCR clonetype from patients with Omicron breakthrough infection and naïve with booster vaccination.
