# Supplementary figures and images for: Single-cell transcriptome atlas of peripheral immune features to Omicron breakthrough infection under booster vaccination strategies
Source: Front Immunol. 2025 Jan 6;15:1460442. doi: 10.3389/fimmu.2024.1460442 (PMC11743671; doi:10.3389/fimmu.2024.1460442)

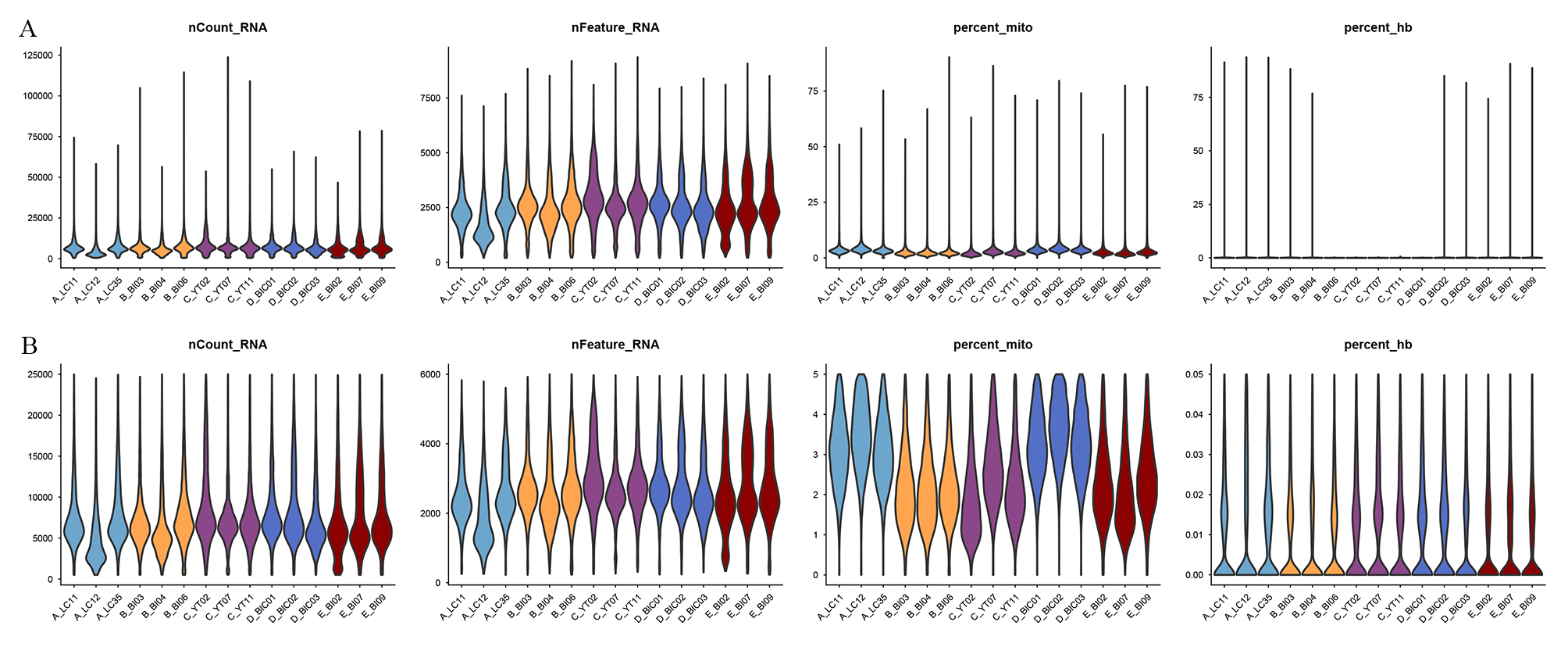

Supplement: Supplementary Figure 1 — Basic characteristics of the integrated dataset. (A) The count, feature, mitochondria percentage, and red blood cell percentage characteristics of 153,395 cells from 15 samples have been sequenced. (B) The count, feature, mitochondria percentage, and red blood cell percentage characteristics of 123,531 cells after QC [file Image1.tif]

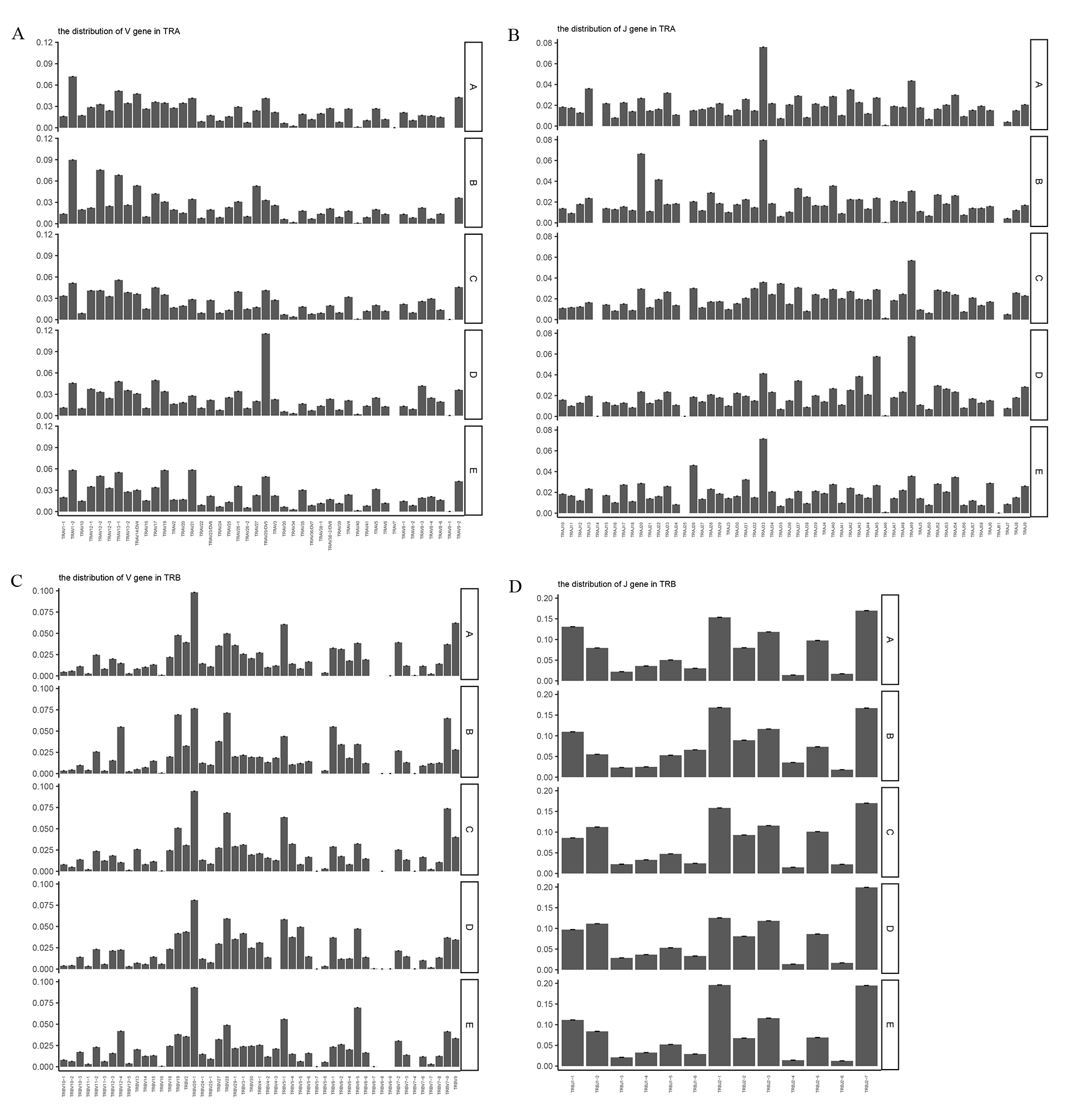

Supplement: Supplementary Figure 2 — The distribution of V/J gene in TCR. (A) The histogram of the distribution of the V gene in TRA across five groups. (B) The histogram of the distribution of the J gene in TRA across five groups. (C) The histogram of the distribution of the V gene in TRB across five groups. (D) The histogram of the distribution of the J gene in TRB across five groups. [file Image2.tif]

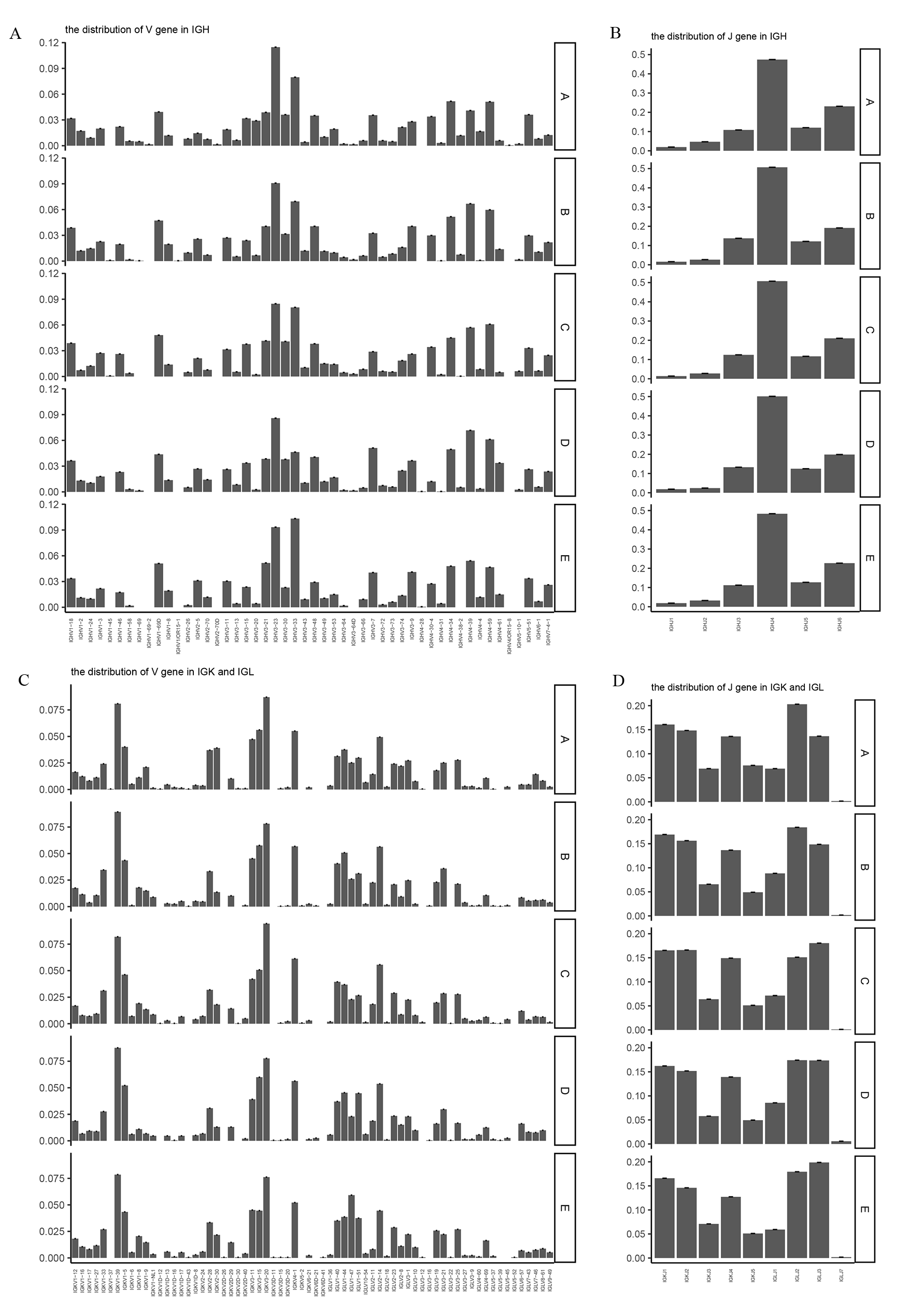

Supplement: Supplementary Figure 3 — The distribution of V/J gene in BCR. (A) The histogram of the distribution of the V gene in IGH across five groups. (B) The histogram of the distribution of the J gene in IGH across five groups. (C) The histogram of the distribution of the V gene in IGL/IGK across five groups. (D) The histogram of the distribution of the J gene in IGL/IGK across five groups. [file Image3.tif]

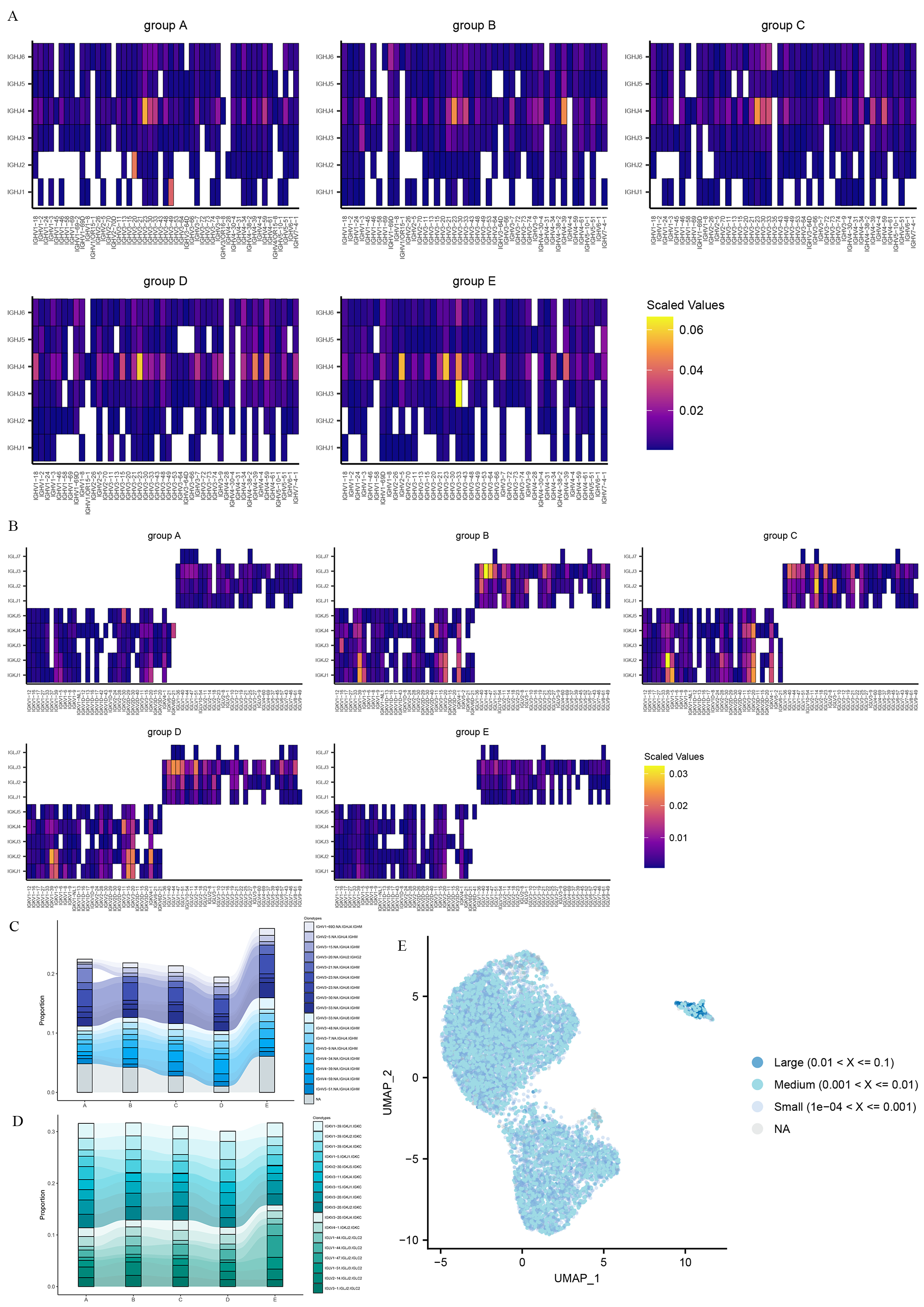

Supplement: Supplementary Figure 4 — The profile of expansion and specific rearrangements of BCR V/J. (A) Heatmap showing the distribution of V/J gene usage in IGH. (B) Heatmap showing the distribution of V/J gene usage in IGL/IGK. (C) The biases and perturbations of the top 10 clones in IGH from patients with Omicron breakthrough infection and naïve with booster vaccination. (D) The biases and perturbations of the top 10 clones in IGL/IGK from patients with Omicron breakthrough infection and naïve with booster vaccination. (E) UMAP projection of the BCR clonetype from patients with Omicron breakthrough infection and naïve with booster vaccination. [file Image4.tif]
